# The barriers and facilitators of HIV-exposed infant testing as perceived by HIV-positive mothers in Botswana: A qualitative study

**DOI:** 10.1371/journal.pone.0273777

**Published:** 2022-08-31

**Authors:** Grace Karugaba, Jennifer Simpson, Bathusi Mathuba, Onkemetse Phoi, Thato Regonamanye, Keofentse Mathuba, Eldah Dintwa, Bornapate Nkomo, Dinah Ramaabya, Mathabo Relebohile Pule, Mogomotsi Matshaba

**Affiliations:** 1 Botswana-Baylor Children’s Clinical Centre of Excellence, Gaborone, Botswana; 2 Global Communities, San Diego, CA, United States of America; 3 Ministry of Health, Gaborone, Botswana; 4 Global Communities, Gaborone, Botswana; 5 Baylor College of Medicine, Paediatric Retrovirology, Houston, Texas, United States of America; Ohio University, UNITED STATES

## Abstract

**Background:**

Despite high rates of HIV testing and enrolment of HIV-positive pregnant women on antiretroviral therapy in Botswana, coverage for HIV-exposed infant (HEI) testing remains suboptimal. Many factors can contribute to suboptimal HEI testing rates, but they have seldom been thoroughly investigated in Botswana. Therefore, the aim of this study was to explore the experiences and perspectives of HIV-positive mothers on the barriers and facilitators of HEI testing to inform interventions to promote HEI testing in Botswana.

**Methods:**

We conducted focus group discussions (FGDs) with HIV-positive mothers who gave birth in 2016 at the three largest public hospitals in Botswana. FGDs were held in Maun, Francistown, and Gaborone from September 2019 to March 2020. The maximum variation sampling method was used to select the participants using information that was abstracted from birth registers and other medical records at the study sites. Mothers were asked to describe their HEI testing experiences, what made it easy or difficult for them to return the HEI for testing, and what needs to be done to improve HEI testing in Botswana. A thematic approach was used to analyse the data.

**Results:**

Fifteen FGDs with 142 mothers (aged 21–52 years) were held. Participants identified several facilitators to HEI testing, including a mother with adequate knowledge of PMTCT, intensive tracking of HEI by healthcare workers (HCWs), positive attitudes of HCWs toward clients, and social support from significant others. Staff shortages at health care facilities, frequent stock-outs of HIV test kits, fear of stigma, fear of positive test results for the child, and transportation challenges were identified as key barriers to HEI testing. Increasing staffing at healthcare facilities, having adequate supplies of HIV test kits, enhanced HEI tracking, easing access to HEI testing services in rural areas, and providing quality PMTCT education were among the proposed interventions to promote HEI testing.

**Conclusion:**

Optimizing HEI testing in Botswana will require multi-level interventions at the policy, health system, community, interpersonal, and individual levels.

## Introduction

Globally, increased access to antiretroviral therapy (ART) has improved the health, quality of life, and life expectancy of people living with HIV (PLHIV) and has significantly reduced the risk of Mother-to-Child Transmission of HIV (MTCT). These advancements have improved the reproductive health of HIV-infected women and the proportion of HIV-infected women who become pregnant has increased over time [1–3]. As a result, promoting the health and quality of life of HIV-exposed infants (HEI) has emerged as an important component of HIV programming.

Botswana’s Prevention of Mother-to-Child Transmission of HIV (PMTCT) program began in 1999, and by 2001, all public health facilities in the country were providing PMTCT services. PMTCT services are integrated into routine Maternal and Child Health (MCH) services and decentralized to the lowest levels of the healthcare delivery system, such as clinics and health posts. According to the Botswana Global AIDS Response Report for 2014, PMTCT services were available in all 634 health care facilities that provided MCH services [4]. In Botswana, a high proportion of women seek antenatal care (ANC), give birth in health facilities, and receive postnatal care (PNC), all of which are free of charge in public health facilities. For example, in 2019, of the 58, 838 women who gave birth in Botswana, 94.3% attended at least one ANC visit, 94.3% delivered in a health facility, and 62.8%attended at least one PNC visit [5]. These statistics show that the majority of pregnant women and new mothers in Botswana remain in contact with the healthcare system and have access to PMTCT services.

The Botswana PMTCT guidelines recommend that all pregnant women seeking ANC at any health facility be routinely tested for HIV unless they opt-out [6]. As a result, many HIV positive women are identified during the ANC period and started on ART. Those who do not attend ANC are tested during labor and delivery, as well as post-partum. Currently, all HIV-positive pregnant women are treated with triple ART regardless of CD4 cell count [6]. HIV positive women are provided with ANC and PNC health education, which includes information on ART for mothers and infants, infant feeding choices, and HEI testing. The guidelines recommend HEI testing using DNA polymerase chain reaction (PCR) tests from dried blood spots (DBS) collected at 4 to 6 weeks of age, coinciding with the mother’s first PNC visit or the child’s first child welfare clinic (CWC) visit, depending on whether the baby is classified as high risk or low risk. Collected samples are transported to a laboratory for processing and results are not available immediately [6]. For breastfed children, a second PCR test is administered 6 weeks after weaning. For infants who test negative at 4–6 weeks, an additional test should be conducted at 18 months of age using rapid antibody testing, with results available within 15 minutes. Infants who test positive at any stage of the care continuum are referred and enrolled on treatment at ART clinics. HIV-positive mothers who opt for formula feeding receive a year’s supply of infant formula which is collected monthly from public healthcare facilities [6].

The Botswana PMTCT program continues to achieve high HIV testing coverage and enrolment of HIV-positive pregnant women on ART. In 2021, the World Health Organization (WHO) certified Botswana as the first high-burden country to achieve the “silver tier status” which moves it closer to elimination of MTCT [7, 8]. According to the National PMTCT Program data for 2020, for example, a high proportion of pregnant women who attended ANC (98%) were tested for HIV or knew their HIV status (only 32% of women knew their HIV status before registering for ANC). The HIV positivity rate among pregnant women was 22%, enrolment on ART was 98%, and the national MTCT rate was 0.56% [9]. Despite those very high rates of HIV testing and enrolment of HIV-positive pregnant women on ART, program data show that coverage for HEI testing at 4–6 weeks was at 83%, falling short of the target of testing all (100%) HIV-exposed children [6, 9]. These statistics show that HEI testing remains a challenge for Botswana’s PMTCT program, and that concerted efforts are required to improve it so that no child is left behind without testing.

Early Infant Diagnosis (EID) allows for the early identification of HIV-infected children, allowing them to be enrolled in ART and reducing morbidity and mortality. EID also helps in infant feeding decisions and alleviates unnecessary fear and stress for mothers and families [10, 11]. Studies conducted in various settings globally have found that infants with early HIV diagnosis and ART enrolment have a higher chance of survival [10, 12–14]. A follow-up study of HEI in Botswana between 2005 and 2012 found that death rates among HIV-positive infants who did not receive ART were significantly higher than those who did [15]. Furthermore, Violari et al. found in a study on children on ART in South Africa that early HIV diagnosis and early ART enrolment reduced early infant mortality by 76% and HIV progression by 75% [11]. This implies that HEI who do not test or receive their test results at the recommended time miss the benefits of EID and enrolment in ART. Therefore, even though the rate of MTCT in Botswana has declined significantly, the promotion of EID remains critical to optimizing the health outcomes and survival for HIV infected infants.

The National PMTCT program has identified low uptake of PNC by mothers as one of the factors contributing to the HEI testing deficit, because babies are not brought for PNC which is the ideal time for them to take their first test at 6weeks [9].Previous research in Botswana has identified barriers to HEI testing such as a lack of a linked medical record system to track HEI nationally [16, 17], mothers’ fear of disclosure to their partners and inability to elicit their support in testing the child [18], mothers’ fear of the child testing HIV positive [19], fear of stigma [20], and the fact that new mothers commonly return to their home villages after giving birth, leading to HEI being lost to follow-up [15]. However, almost all the previous studies were quantitative in nature [15–19]. To our knowledge, one qualitative study only included 21 HIV positive mothers from Gaborone [20]. As a result, little was known about the scope and nature of the barriers and facilitators to HEI testing perceived by the HIV-positive mothers in Botswana.

The Ministry of Health (MOH) and its partners have made significant efforts to promote PMTCT program uptake in Botswana, including increasing the number of laboratories that can perform PCR tests, training nurses at all levels of the health care system to collect dried blood spot (DBS) samples for PCR testing and to initiate prophylaxis and lifelong ART [7, 9]. Community-based partners have been engaged in the follow-up of mothers and their children and encouraging them to return to health care facilities for PNC, CWC, and HEI testing [4]. Resultantly, HEI testing rates have increased over the years, for example, rising from 57% in 2017 to 83% in 2020 [7, 9]. Thus, it is important to examine how HEI testing needs are currently being met as well as what challenges and obstacles may still be impeding the uptake of HEI testing services in Botswana. This study focused on the perspectives and experiences of HIV positive mothers on the facilitators and barriers to HEI testing. The objective of the study was to give a voice to HIV-positive mothers, allowing their stories and views to inform the promotion of HEI testing in Botswana.

## Materials and methods

### Study design, setting and sample

This was a qualitative study that used focus group discussions (FGDs) with HIV positive mothers who delivered children in 2016 at three large public hospitals in Botswana: Princess Marina Hospital in Gaborone, Nyangabgwe Referral Hospital in Francistown, and Letsholathebe Memorial Hospital in Maun. The three hospitals are the largest birthing sites in Botswana, accounting for more than half of the country’s annual deliveries. The three hospitals serve as referral points in the healthcare delivery system, with large catchment areas that include urban, rural, and hard-to-reach areas. In this study, a maximum variation sampling strategy was used to ensure a wide variety of HIV-positive women were included in the sample. To achieve this, the researchers used the social-demographic and clinical information from medical records to select cases representing variation on dimensions of interest, such as place of residence, age at delivery, ANC enrolment by trimester, VL levels, as well as mothers who did and those who did not return their children for testing at the recommended time (6 weeks or 18 months). On average 8 to 12 mothers attended each FGD. The final FGD sample size (142) was determined by data saturation.

### Inclusion and exclusion criteria

Participants included mothers with a documented HIV-positive status in the birth register, who gave birth at Nyangabgwe Referral Hospital, Letsholathebe Memorial Hospital, or Princess Marina Hospital in 2016, and who gave consent to participate in the study. Excluded from the study were HIV-positive mothers who did not consent to participate in the study, as well as those who lived very far from the data collection sites (over a radius of 100 kilometres).

### Data collection process

The standardized open-ended FGD guide using a set of open-ended questions was developed and used by the researchers. The development of the FGD questions was based on a detailed literature review on factors that affect HEI testing and consultation with HCWs at the study sites. The FGD guide was written in English, and during the sessions, the bilingual facilitators read the questions in English and translated them into Setswana the native language of the participants. When answering questions, participants had the option of using Setswana or English. Audio of responses was recorded, and written notes were taken. The questions in the focus groups covered the following broad topics: knowledge of PMTCT and HEI testing; experienced barriers and facilitators to HEI testing; and recommendations on what should be done to improve HEI testing in Botswana. The FGD sessions lasted approximately 2 to 2.5 hours on average. All FGDs were facilitated by a consistent team of three researchers with extensive experience in qualitative research.

All the FGDs were held at Botswana-Baylor Children’s Clinical Centre of Excellence (Botswana-Baylor) in Gaborone, Nyangabgwe Referral Hospital in Francistown, and Letsholathebe Memorial Hospital in Maun. The FGD participants were reimbursed for transportation costs based on actual travel costs, with a minimum of 30 Pula (3 United States Dollars).

### Study ethical considerations

Written informed consent was obtained from each HIV-positive mother who participated in the FGDs. Consent forms were available in both English and Setswana. Due to the private nature of the discussions in FDGs, the researchers assured participants of strict adherence to data protection and confidentiality. All FGD transcripts were de-identified by removing any information that could compromise the privacy of participants. All focus groups were held in rooms that provided the participants with privacy and comfort.

Ethical approval for this study was granted by the Institutional Review Board (IRB) of Global Communities (PCI Protocol #24), Botswana-Baylor (BBCOE-IRB-1901-15), and the Ministry of Health and Wellness (HPDME 13/18/1). The research was also approved by participating sites internal IRBs, that is, Ngamiland DHMT IRB (M5/50/2), Nyangabgwe Referral Hospital IRB (NRH 1/2/17), Princess Marina Hospital IRB (dated: 19/03/2019), and Greater Gaborone DHMT IRB (GGDHMT 2/27VI (4)). The managers of the hospitals where the data was collected gave administrative approval for the study to be conducted in their facilities.

### Data management and analysis

Basic statistical analyses were performed to compute frequencies and percentages from socio-demographic and clinical data about the FGD participants to provide context for the perspectives presented in the study. The FGDs were audiotaped and then transcribed verbatim. Thematic analysis was used to analyze the qualitative data generated from the FGDs. This was done by examining, interpreting, and categorizing the participants’ narratives as facilitators, barriers, and recommendations for improvement to HEI testing. Lastly, the categories were grouped together under overarching themes and mapped to the social-ecological framework [21]. The themes included those that emerged inductively from the FGD data as well as themes that were informed by pre-existing literature on determinants of HEI testing.

The data generated by FGDs showed very close similarities in experiences and world views of participants hence making it easy to categorize the data into themes. Extensive quotations to be used in reporting the results were identified to keep the issues expressed by the participants clear and concise rather than abstract. The researchers conducted three sequential analysis rounds until no new information or themes were found. The researchers subjectively determined that saturation had occurred after the completion of 15 FGDs.

## Results and discussion

The purpose of this study was to investigate the perspectives of HIV-positive mothers on the barriers and facilitators to HEI testing, as well as what needs to be done to improve HEI testing in Botswana. The study’s findings are presented and discussed in this section, following the levels of the social-ecological framework.

In total, 142 HIV-positive mothers (aged 21 to 52 years) participated in 15 focus groups in Gaborone (48.6%), Francistown (29.6%), and Maun (21.8%). Table 1 shows the study population’s socio-demographic and clinical characteristics. The participants lived in rural (49.3%) and urban (50.7%) areas. In terms of occupation, 5.6% worked in professional cadres, 46.4% worked in elementary jobs, and 48% were unemployed. During the index child’s pregnancy, the mothers enrolled in ANC during the first trimester (56.3%), second trimester (40.8%), and third trimester (4%).

**Table 1:** Social-demographic and clinical characteristics of the sample (n = 142).

FGD participants per site n (%)	^Number of FGDs^	Number of participants
Princess Marina Hospital	6	69 (48.6%)
Nyangabwe Referral Hospital	5	42 (29.6%)
Letsholathebe Memorial Hospital	4	31(21.8%)
**Age range at study visit (years)**	21–52	
**Education level at study visit n (%)**		
Primary	9 (6.3%)	
Secondary	98 (69%)	
Tertiary	17 (11.9%)	
Other (post-secondary training courses)	18 (12.7%)	
**Occupation at study visit n (%)**		
Professional	8 (5.6%)	
Elementary	66(46.4%)	
Unemployed	68 (48%)	
**Residential area at study visit n (%)**		
Urban	72(50.7%)	
Rural	70 (49.3%)	
**CD4 count (cells/mm** ^**3**^ **) at study visit n (%)**		
<300	10 (7%)	
300–500	22 (15.5%)	
	96 (67.6%)	
>1000	11(7.7%)	
Unknown	3 (2.1%)	
**Viral load (copies/mL) at study visit n (%)**		
<400	141 (99.3%)	
Unknown	1 (0.7%)	
**HIV status of partner at study visit n (%)**		
Negative	83 (58.5%)	
Positive	59 (41.5%)	
**Age at delivery in 2016 n (%)**		
≤35years	99 (70%)	
>35years	43(30%)	
**ANC enrolment n (%)**		
1^st^ Trimester	80 (56.3%)	
2^nd^ Trimester	58 (40.8%)	
3^rd^ Trimester	4 (2.8%)	
**Mode of infant feeding n (%)**		
Formula feeding	110 (77.5%)	
Breastfeeding	29 (20.4%)	
*Sequential feeding	3 (2.1%)	
**HIV-exposed infant testing n (%)**	**YES**	**NO**
≤6 weeks	130 (91.5%)	12 (8.5%)
18 months	113 (79.6%)	29 (20.4%)

According to the mothers, HEI testing was influenced by factors at the individual, interpersonal, community, health-care system, and policy levels. The mothers proposed multi-level actions to promote facilitators and address barriers to HEI testing. Table 2 summarizes the barriers, facilitators, and recommendations aligned with the social-ecological framework.

**Table 2:** Summary of HIV positive mothers’ perspectives on facilitators, barriers and recommendations to promote HIV exposed infant testing.

LEVEL	BARRIERS	FACILITATORS	RECOMMENDATIONS
**POLICY**	• The HIV testing policy that requires only a parent or legal guardian to consent to child testing resulted in delayed testing in cases where the mother or legal guardian was away	**-**	• ^a^HCWs should be flexible to allow secondary caregivers to consent to HEI testing or to receive test results (where a parent or legal guardian is not available to consent)
**COMMUNITY**	• Living in remote and rural areas and the long distance to a health facility that offers HEI testing services • Fear of stigma and discrimination in the community	• Availability of peer support in the community • Follow-up of mother-infant pairs by community health care workers, and other service providers	• Bring ^b^HEI testing services closer to the people in remote and rural areas • Increase community education on HEI testing and reduce stigma around HEI testing • Consider home-based testing and other mobile outreach activities • Integrate HEI testing with other community-based services for mothers and their children
**HEALTH SYSTEM**	• Understaffing at health facilities affects service delivery, e.g. the quality of ^c^PMTCT health education • Frequent stock-out of HIV test kits and other laboratory supplies • A limited number of HCWs have been trained to collect ^d^DBS blood samples • The long turnaround time for DNA PCR test results leading to multiple visits to the clinics by mothers • Perceived negative attitudes of some HCWs towards patients • The lack of a linked e-register to track HEI nationally	• Quality PMTCT education and counselling provided by HCWs • Intensive tracking and follow-up of mother-infant pairs by HCWs • Positive attitudes of HCWs towards the mothers • HEI testing integrated with other child health care services (one-stop-shop model) • Opportunity for socialization and networking with peers at the health facility	• Ensure adequate supplies of HIV test kits at health facilities • Increase staffing at health facilities to improve service delivery • Improve the quality of PMTCT health education provided by HCWs • Train and equip HCWs with good customer care skills • Enhance facility and community-based tracking of mother-infant pairs • Integrate HEI testing with other child health care services to reduce the number of visits to the clinic, and to counter the stigma associated with identifiable HIV services
**INTERPERSONAL**	• The frequent change of caregivers for the HEI • Fear of disclosure, stigma and discrimination • Lack of support from a spouse, family member, or friend/peer	• Availability of social support from a spouse, family member, or friend/peer	• Provide counselling to encourage partner, family or peer involvement in HEI testing. • Link HIV positive mothers to peer support
**INDIVIDUAL**	• Mother’s fear of an HIV positive test result for the infant • Lack of self-acceptance of HIV status by the mother • Limited knowledge of PMTCT and HEI testing • Limited knowledge of children’s rights • Child negligence • Mother or caregiver forgetting the child’s testing appointment	• A mother having adequate knowledge of PMTCT and HEI testing • The mother’s acceptance of her HIV status • A mother’s innate desire to protect the health of her child • Good ^e^ART adherence during pregnancy and after delivery	• Educate mothers on children’s rights • Set up reminders to avoid missing HEI testing appointments • Empower mothers to exercise self-responsibility and self-motivation to take their children for testing appointments • Support mothers to accept their HIV-positive status and learn to live with it • Mothers should utilize peer support at health facilities or in the community

^a^ HCW-Health Care Worker

^b^HEI-HIV exposed infants

^c^PMTCT-prevention of mother-to-child-transmission of HIV

^d^ DBS-Dried Blood Spot

^e^ART, antiretroviral therapy

### Policy level factors

#### The HIV testing policy that requires a parent or a legal guardian to consent for the child to be tested or to receive test results

Some mothers identified the HIV policy, which requires consent from a biological parent or legal guardian to test or collect test results, as a barrier to HEI testing. In some cases, where the mothers or legal guardians were not available to provide consent for various reasons (such as being too busy at work or school, being ill, or having travelled), the healthcare workers refused to test the HEI who were accompanied to the testing appointments by other secondary caregivers (grandmothers, aunts, uncles, older siblings, other relatives, maids, etc.).To address this barrier, the mothers called for flexibility in the HIV testing policy so that secondary caregivers with delegated responsibilities for the child can consent to testing, to allow early identification of those who are HIV positive and timely enrolment on ART to reduce morbidity and mortality. Mothers recommended that HCWs should allow the child to be tested if there is evidence that the parent or legal guardian has permitted the secondary caregiver to take the child to the clinic for a testing appointment.

*“Yes*, *for me*, *what disappointed me was that my child was staying with my grandmother*. *My grandmother would go to the local clinic where they were staying and get infant formula for the child*. *She had the clinic card*. *When it was time for the baby to be tested*, *they said that I needed to be present for the child to be tested*. *I was perplexed as to why they needed my presence when the clinic card contained all of the necessary information*, *and they had been issuing infant formula to my grandmother without my presence*.*”*

*“If you don’t have a problem*, *and you tell them you don’t mind the child being taken to the testing appointment by another person*, *they tell you that they are not supposed to disclose the child’s HIV status to other people*. *I couldn’t understand because I had disclosed to my mother and given her permission to take my child for testing*. *How could I turn around and sue them for that*? *"*

*“I propose that for us mothers who are working to provide for our families*, *the clinics should allow those who are caring for our children to bring them for testing on our behalf*. *That will make our lives easier*.*”*

This phenomenon is most likely the result of service providers and parents/guardians misunderstanding and/or misinterpreting the law and the HIV testing policy. According to the Botswana National Policy on HIV and AIDS (2012), section 5.3, children aged 16 years and above can consent to their own testing, while children below that age can be tested with the consent of a parent or legal guardian [22]. The Botswana Children’s Act of 2009 defines a parent as "a biological parent, adoptive parent, foster parent, or step-parent" [23]. The Children’s Act also defines a guardian as "a person who has charge or control of a child or a person appointed by law to be the legal guardian of the child”. The National HIV and AIDS policy goes further to state that "if the individual cannot obtain consent from a parent or legal guardian, an HIV test may be administered provided a medical practitioner determines the need for such a test". Therefore, a combination of these two instruments should provide guidance in all possible scenarios, ensuring that no child is left behind.

These findings highlight the need to train or retrain HCWs on the existing law and policy regarding HIV testing for children, as well as providing them with guidance to enable them to resolve specific situations involving HEI testing consent [24]. This would be consistent with the United Nations Convention on the Rights of the Child, which affirms the child’s right to "survival, life, and development" (Article 6), as well as the right to health (Article 24) [25].

### Community level factors

#### The fear of stigma and discrimination

Many mothers said that the fear of stigma and discrimination in the community and health facilities was a significant barrier to HEI testing. The mothers said they were mostly afraid of stigma and gossip from community members at the health facilities. The mothers did not want to be seen around HIV testing rooms in the local clinics because they feared, not only stigma against their children, but also against themselves since HIV testing for children indicates maternal HIV status. The mothers described how HIV testing rooms at health facilities, which are easily identifiable with HIV posters or other indicators, compromise confidentiality. Some mothers reported that they did not attend the 18-month test appointment after their children tested negative at 6 weeks to avoid multiple encounters with stigma from community members at health facilities.

*“Sometimes when you go to the clinic*, *you will find someone from your neighbourhood*, *maybe your neighbour*, *who didn’t know that you are HIV positive*. *She or he will start gossiping in the community*, *"I saw her testing her child at the clinic*. *She has HIV*.*" So this means you won’t even be free in your own house because the neighbour saw you at the health facility*. *Again*, *having to go back for another test at 18 months is a challenge*. *People don’t want to be seen going back to the testing room*. *I wish testing could be done only once*. *Maybe that is why a lot of mothers test the children at 6 weeks only*. *"*

However, some mothers in the FGDs, particularly those who tested their children at the recommended time, said they had accepted their HIV status and had developed the courage to counter stigma and to prioritize their children’s health.

*"I was formula-feeding*, *so coming to the clinic to get formula helped me to be bold and confident about my status; I didn’t care what people thought of me*, *I only did what was best for my child*.*"*

*"I don’t care what people say about me anymore*. *I come from a small village*. *People there like gossiping about HIV-positive people*, *and whenever they see me at the clinic*, *they stare at me*, *trying to embarrass me*. *I just ignore them*, *but I know it’s not easy for other young mothers*. *They always envy me*, *to the extent of asking me how I manage*.*"*

To reduce stigma and increase HEI testing, the mothers recommended that all HEI testing services be fully integrated with other child health care services (e.g., PNC, immunization, and monthly weight monitoring) so that people do not know the mother’s or infant’s HIV status based on the services they line up for. The mothers also proposed that counselling or health education be provided to assure mothers of confidentiality, and children’s HIV testing information should be kept in an electronic information system rather than on the CWC card which is easily accessible to other people.

*“The HIV test kits should be in every consulting room in the clinic so that testing can be done without having to go to the HIV screening room*. *I don’t like a system where testing is done in that room with a poster*. *The testing rooms can also be swapped daily so that the people who came to the clinic would not know where the HIV testing room is exactly*.*”*

*“It is challenging for the 18-months test because you have to queue with all the other patients*. *When you are queuing with a baby*, *there is a lot of stigma*, *and people stare at you and make comments*. *So just like the 6-weeks test*, *there should be a safe place for testing babies at 18 months*. *For the 18-months test*, *let us use the same room where the 6-weeks testing is done*."

These findings are consistent with other studies conducted in various African settings, which found that fear of stigma in healthcare settings negatively influenced the uptake of PMTCT services, including HEI testing [26–29]. The findings emphasize the need for the MOH to strengthen measures to offset HIV-related stigma in health facilities, such as integrating all HEI testing processes with other child health care services to protect mother-infant pairs from stigma. Several other studies have found that integrated service delivery reduces stigma in healthcare settings [30–32]. Furthermore, the results highlight the need for MOH (in collaboration with community-based partners) to enhance community sensitization and education to reduce stigma and foster a supportive environment for HIV-positive mothers and their children.

#### The challenges of living in rural areas

Living in rural areas (for example, cattle posts, farms, settlements, and other hard-to-reach areas), and having to travel long distances to reach health facilities that offer PMTCT services was identified as a barrier to HEI testing. The mothers who lived in rural areas reported financial challenges (they could not afford transportation) and unreliable means of transport to health care facilities. Other barriers to HEI testing reported by mothers from rural communities included: limited information on PMTCT; understaffing at health facilities (they could afford to get to the clinic once but couldn’t afford to return after being turned away); fear of stigma because everyone in the community knew each other, making concealing one’s HIV status difficult; and late or non-enrolment in ANC and PNC, leading to missed opportunities for health education and HEI testing.

*“The ambulance (mobile clinic) only comes once a month on the 25th; if you don’t have transportation money on the 25th that will be it*. *You can’t walk to where the ambulance stops because it’s too far away*. *Again*, *transport is unreliable; sometimes you won’t find any car*. *So you will attend the clinic the following month if you manage to keep that money for transportation*.*”*

*“Talking about rural areas*, *I am from a rural area*. *The biggest problem that we are having in our health post is a shortage of staff*. *The one or two nurses who are in the health post cannot even do a home visit if people default from ART*. *Therefore*, *tracking mothers to their homes to test their children will be impossible*. *They cannot even phone you to remind you of your 6 weeks PNC review because they are always busy*.*”*

The mothers recommended that the Government of Botswana should accord high priority to rural settings by bringing services closer to the people in those areas. Provision of transport support for mothers who have to travel long distances for HEI testing appointments, home based testing, and other community outreach activities are some interventions to be considered. Many mothers also advocated for a consolidated or one-stop-shop service delivery model which would combine, multiple child health care services in one location and on one day, reducing clinic visits and transportation costs for mothers.

*“I believe the government should create a program that includes every service for infants so that on the day for a check-up*, *you receive all services in one day and in one place rather than being asked to come back another day for another service that could be provided on the same day*. *"*

Other research has found that high transportation costs and unreliable modes of transportation increase attrition from PMTCT and other ART programs [26–29]. The Government of Botswana provides a primary health care system that offers essential health services in an evenly accessible way to all people in Botswana. Nationally, 95% of the total population (89% of the rural population) lives within eight kilometres (km) of a healthcare facility [33]. As a result, HIV testing, treatment, and care services are widely available and free of charge in both rural and urban areas. Furthermore, PMTCT is available in all 634 health facilities that provide MCH services in Botswana [4]. Despite this significant progress towards universal health care and PMTCT coverage, the findings of this study indicate that more needs to be done to ensure women in rural settings have easy access to HEI testing services.

The eight km distance to a health facility, in particular, may be too far for a new mother with a young child to walk and would necessitate the use of some mode of transportation. Furthermore, women who live in hard-to-reach areas (more than eight km) may face additional challenges and require targeted attention. Therefore, providing transport support to mothers as well as expanding access to HEI testing services at local clinics, health posts, and mobile stops, as well as through home based testing and other outreach services should be prioritized. Government programs and other stakeholders that serve rural communities should prioritize interventions aimed at improving access and utilisation of PMTCT and HEI testing services.

As a starting point for responding to mothers’ call for consolidated child health care service delivery, health facilities should coordinate all HEI testing with other CWC appointments (PNC, immunization, monthly weight monitoring, infant formula supplies, etc.) so that mothers do not have to return to the health facility for multiple appointments.

### Healthcare system or institutional level factors

In this study, health care delivery issues were the most commonly cited facilitators and barriers to HEI testing uptake.

#### Tracking and follow-up of HEI by HCWs

Intensive tracking and follow-up of mother-infant pairs in health facilities and in the community by HCWs was identified as a major facilitator for HEI testing. Many mothers reported that follow-ups and reminders by HCWs motivated them to bring their children for testing at the recommended time. The mothers reported that HCWs contacted and reminded them about their child’s testing appointment via phone calls, text messages, and home visits. In addition, the mothers said that attending the 6-week PNC visit and other CWC appointments (immunization, monthly weight monitoring and infant formula collection) enabled HCWs to screen the CWC card, identify, and refer HEI for testing.

*“I only tested my child at the ages of six weeks and eighteen months*. *I tested my child on time and the results were just fine*. *Maybe again it was the fact that where I came from*, *the HCWs will follow you up*. *They will remind you to take your child for testing prior to the due date*. *If you miss the testing appointment*, *they will come to your home to ask you to go to the clinic and have the child tested*.*”*

*“I just want to encourage other clinics to benchmark on what is done in the clinic where I go*. *In the clinic where I go*, *when the baby is supposed to be vaccinated or tested*, *the nurse does not weigh the baby before checking everything*. *The nurse would first check through the baby’s CWC card and ask the mother to go and have the baby vaccinated or tested before coming to take the weight*. *In that way*, *the babies never skip the vaccines or testing because you cannot weigh the baby before doing all those*.*”*

However, some mothers observed that follow-up by health care workers in 2016 was not as intense as it had been in the early years of the PMTCT program, and they recommended that it be improved. The mothers advocated for intensified follow-up of HEI through SMS messages, phone calls, social media, and home visits. The mothers recommended that all HIV-positive mothers should be sent reminders of their child’s testing appointments as a standard procedure. It was also suggested that information about HEI testing should be added to the computer system that is used to track PLWH on ART, or that a separate computer system be developed to facilitate the tracking of mother-infant pairs nationally. There should be a list of all HEI that need to be accounted for by the health care system.

*"There is a child I delivered in 2012*. *At that time*, *HCWs were phoning us and asking if we had given the baby the medicine and if we had done anything else*. *That follow-up in and of itself encouraged me to do what was best for my baby*. *However*, *when I gave birth in 2016*, *I did not see it happening like it did in 2012*. *"*

*"All the babies and their mothers should be followed-up*, *for example*, *if so and so lives in a particular village*, *HCWs in that area can check on the mother’s condition*. *When mothers bring their babies in for monthly weight monitoring*, *the CWC cards should be checked*. *All hospitals should notify the clinics about the mothers with children who need to be followed-up and tested*.*"*

*"Furthermore*, *I recommend testing services be brought to the homes of those mothers*. *So they may go from house to house*, *testing these children*. *This would lessen the excuses of mothers who would say*, *"I was not able to go to Molepolole for testing because of transport problems*.*" If they are unable to help me with transportation*, *then they may simply come to my home and test the child*. *"*

The mothers also suggested that health care facilities establish multiple service points by incorporating HEI testing into all other child health care services. It was noted that most mothers bring their children to health facilities for PNC, immunizations, and monthly growth monitoring. As a result, the majority of children remain in contact with the healthcare system. Therefore, HCWs should screen and identify all children who need to be tested. HCWs should make sure that mothers do not leave without testing their children. However, all tracking methods must ensure that the mother’s and HEI’s privacy is protected.

*“The HCWs should also take advantage of checking if the child is tested whenever they come across them in the clinic*. *If the child is brought in for any form of sickness or monthly weighing*, *if not tested*, *the child should be tested there and then*. *They should make sure that by the time the child leaves the clinic everything has been done*.*”*

These findings suggest that expanding HEI testing in Botswana beyond current levels could benefit from intensified tracking of mother-infant pairs via various interventions, such as fully integrating HEI testing into other child health care services; reminding all HIV-positive mothers of their child’s testing appointment via telephone calls or text messages; and establishing a nationally linked electronic register to track mother-infant pairs. This finding is consistent with previous studies conducted in a variety of research settings globally which found that intensive tracking efforts by HCWs were effective in increasing uptake of HEI testing [34–37]. Furthermore, the findings highlight the need to expand community-level tracking of HIV-positive mothers and their children. According to the Botswana National Guidelines for Implementing Integrated Community-Based Services, deliberate efforts should be made to engage and or strengthen the capacity of community-based partners in tracking mother-infant pairs to return to the health facility for HEI testing and test results collection [38].

#### The attitudes of HCWs towards mothers

The positive attitudes and behaviours of HCWs towards mothers facilitated HEI testing. The FGD narratives show that some HCWs went out of their way to support mothers and encourage them to return the HEI for testing at the recommended time. Most of the mothers in the FGDs described how easily they interacted and communicated with the HCWs on a variety of clinical and psychosocial issues. Those mothers said they were encouraged to bring their children for testing because the clinic environment was friendly and supportive.

*“For me*, *the first nurse that was counselling me was very helpful*, *I became so free to continue with the services*, *taking my medicines during pregnancy*, *and testing my child on time*. *She made my path easy*, *everything became easy*, *and she gave me a lot of hope that sustained me throughout the testing process*.*”*

*“I only received counselling from the nurse who started me on treatment*. *I told her everything about my life*, *how I lived*, *and how I got infected with HIV from my new partner*. *She encouraged me and gave me hope*, *saying that I should be strong*, *do the right thing and follow the right steps*. *These few words of encouragement helped me to make the right decisions*.*”*

Conversely, the negative attitudes and behaviours of some HCWs towards the mothers were a barrier to HEI testing, particularly when the mothers missed HEI testing appointments or did not follow other clinic-provided child health care instructions. To overcome this limitation, many mothers across all the FGDs strongly advocated for the government to train and equip HCWs with good customer service skills (more welcoming, less rude, more respectful, more understanding, and stop shouting at patients). More mothers would be encouraged to return their children for testing if the environment was welcoming and dignified.

*“Another thing is the attitude of HCWs*. *I always hear people saying that “I don’t think I will come back to the clinic*, *the nurses here are rude”*. *So if the HCWs could change their negative attitudes toward clients*, *maybe more mothers will come back to have their children tested*.*”*

The findings are consistent with previous research conducted in diverse settings, which found that positive HCW attitudes facilitated HEI testing [28, 29, 39, 40]. The findings call for increased HCW training on how to improve provider-patient relationships and maintain a respectful, welcoming, and dignifying health facility environment, which improves patient care quality and HEI testing uptake.

#### The shortage of healthcare workers who are trained to administer HEI testing at healthcare facilities

A shortage of HCWs who were trained on dried blood spots collection was identified as a barrier to HEI testing. Some mothers said that they took their children to the health facility for testing but were turned away and told to come back another day because the nurse who was trained to take blood samples from HEI was not available. As a result, some mothers never returned to the clinic for rescheduled appointments. Having enough HCWs who are trained to obtain blood samples from children would improve service delivery and reduce transportation costs and waiting time for mothers, they said.

*"At the clinic where I go for my check-ups*, *when I wanted to test my child*, *they told me there was no staff who does child testing*. *At that time*, *I was working*, *so I couldn’t go for the next appointment*.*"*

*"I tested my child after 6 weeks because I was told that the nurse who was supposed to test my child was not around*, *so I was given a new date*, *but I did not go back for testing*. *I was simply too lazy to go back to the clinic*. *I also thought they might return me*. *The child was tested recently when those people from Gaborone were here*. *The results were negative*.*"*

*"The Government should provide enough resources and hire a sufficient number of staff to provide services to clients*. *They shouldn’t postpone testing because the nurse who tests children is not on duty*. *It is not acceptable*. *If I ask for permission from work this week*, *I won’t be able to do so next week*. *So if one nurse is not on duty*, *the others should do the testing*.*"*

These findings are consistent with previous research, which found that staff shortages and inadequate HEI testing skills among HCWs are barriers to HEI testing [26–29, 39–41]. The findings highlight the need for the MOH to increase the number of staff dedicated to providing PMTCT services at all health facilities to improve service delivery. Training on DBS sample collection should be conducted on a large scale to enable a wider cadre of HCWs to provide HEI testing services.

#### Frequent stock-out of HIV test kits and the long turnaround time for DNA PCR test results

The most frequently reported barriers to HEI testing were appointments being rescheduled due to a shortage of HIV test kits and other laboratory supplies, and the prolonged turnaround time for PCR test results. Many mothers went to health facilities to have their children tested, but they were told to come back another day because there were no HIV testing kits. Furthermore, some mothers reported that they waited for a long time for their child’s PCR test results. Mothers had to return to the clinic several times because the results weren’t ready when they were told they would be, because the results were either not back from the laboratory or were lost or unclear, requiring the child to be tested again. The mothers experienced a great deal of stress, anxiety, and frustration as a result of delays in accessing testing services and the slow turnaround time for results. As a result, some mothers did not return to the health care facility for their child’s testing appointment or to receive the test results when they became available.

*"When you arrive at the clinic*, *you are told that the things that are used for testing are not available*. *You are told to come on Thursday*, *and that Thursday you don’t have transport money to go there*. *You end up not going back and not testing your baby*, *as it happened to me*. *I was supposed to go test the baby when he was 18 months old but I ended up testing him when he was 3 years old*, *recently when I was called to come to Botswana-Baylor*.*"*

*“It is emotionally torturing having to wait for so long for the child’s test results; it is stressful and you can’t concentrate on anything*. *At the very least*, *if the results come back quickly*, *as they do for adults*, *you’ll know the child’s status*, *and if necessary*, *start treatment before the child becomes ill*.*”*

*"HIV test kits should always be available*. *For example*, *in some clinics*, *HIV test kits are not always available at 6 weeks; this paper that has circles “DBS card” and you have to go to the clinic again and again without any help*. *And at 6 weeks*, *the test results take a long time to come; I feel they could try to shorten the time it takes for the results to come*.*”*

The WHO recommends that DNA PCR test results for HEI be returned to the facility and to mothers within four weeks of sample collection [42]. Delays in HEI testing or the return of test results to mothers may cause delays in ART initiation and lead to poor health outcomes for HIV-positive infants [15, 26, 29, 43–45]. These findings highlight the critical importance of the MOH and District Health Management Teams (DHMT) reviewing laboratory commodity management and monitoring systems to ensure that enough HIV test kits and other laboratory supplies are available to meet the testing needs of all health facilities. Adoption of best practices in EID, such as point of care HIV testing, [37, 39–41] as well as the use of communication technology for prompt feedback of results from the laboratory to clinics and to the mothers are also models to consider to reduce DNA PCR test result turnaround time.

### Interpersonal level factors

#### Frequent changes in caregivers for the HEI

Many mothers cited the frequent change of a child’s caregivers as a barrier to HEI testing. Some mothers said they moved away from the child due to work, school, or other responsibilities, leaving the child with a secondary caregiver, which often resulted in missed HEI testing appointments. The challenges were: mothers’ failure to disclose to secondary caregivers about the child’s testing appointments; the HIV testing policy, which requires the parent or legal guardian to consent for the child to be tested; and or the child being left with elderly secondary caregivers who either forgot the appointment, were not knowledgeable about PMTCT and HEI testing, or lacked the transport money or energy to take the child to the health care facility for testing.

*"So*, *as I am working here in the city to provide for my family*, *my grandmother is usually told that there is no nurse*, *then she will be asked to return*, *and she won’t have enough money to go to the next village*. *At the same time*, *I am also far away and not in a position to help her because of work*. *I have to work and provide for them*. *This resulted in me testing the child very late*. *"*

Other studies found that HEI whose mothers had died or moved away for various reasons were unable to get tested on time or at all [26]. The findings highlight the importance of HCWs identifying and actively assisting mothers who intend to leave HEI with secondary caregivers as well as providing adequate PMTCT and HEI testing education to those secondary caregivers. This finding supports the mothers’ proposal that HCWs allow secondary caregivers who have been authorised by the mothers to consent to HEI testing.

#### Having social support from family, partner, and or a peer/friend

The FGD narratives showed that strong support from a family member, partner, and or trusted friend/peer, following disclosure of HIV status to them, was a major motivator for HEI testing. The mothers who disclosed their status to significant others were able to freely elicit support and encouragement to return HEI to the health facility for a testing appointment. Supportive family members provided financial support, information about HEI testing, reminders about testing appointments, emotional support, and a safe home environment for the mother and the infant.

*"At home*, *they would say*, *"bring the card and let’s see when you have to go for testing the child*.*"Sometimes they would ask if I had taken the child for testing*. *I would be shocked because I would have forgotten the testing appointment*. *There and then*, *I would leave and take the child to the clinic*. *"*

*"When I delivered*, *my sister was the one helping me at home*, *doing everything for me*. *And she also went with me to test the baby while he was still young to check if he was okay or not*. *The baby is negative and is doing fine*. *With my sister’s help*, *I realized there was nothing to worry about and I continued testing the baby*. *"*

The supportive partner or father of the child reminded the mother about HEI testing appointments, provided financial support, and, in some cases accompanied the mother or took the child to the healthcare facility for testing appointments.

*“I was thankful to God*. *The father of my child is the one who encouraged me to get my child tested*. *He is also on treatment*. *I went with him to the clinic*. *He tested positive and accepted himself*. *He always encourages me*. *At one time we were wanted in the clinic and we went together*. *We do not have any problems*.*"*

*“For testing at 18 months*, *I was working at the time*, *so I had to get my child’s father to take him to the clinic for the test*, *and they assisted him*.*"*

Mothers in the FGDs shared experiences of how they supported their peers (other HIV-positive women) to overcome fears of HEI testing. In some cases, they had accompanied their peers to the health care facility to have their children tested. A few mothers said they anonymously informed health care workers when a peer refused to take her child to the health care facility for testing.

*"My friend and I were both expecting babies at the same time*.*”She was afraid to enrol in the PMTCT program*. *I then joined the program*. *During the check-ups we would go together*, *she ended up joining because I would encourage her to go with me*. *We would go together as friends*. *She ended up feeling encouraged that she had someone to go with*. *Even if our children’s test results may differ*, *we all went through the same path*.*”*

Conversely, some mothers, due to fear of stigma and rejection, did not disclose their HIV status to significant others (family members, partners, peers, or friends) and consequently could not enlist their help in testing the child. The mothers recommended that HCWs should counsel HIV-positive women on how to disclose their HIV status and involve significant others in HEI testing. The mothers advocated for the MOH and community-based organizations to set up peer support groups for HIV positive pregnant women and new mothers at health facilities and in the community.

These results emphasize the importance of HCWs, including a discussion about disclosure of HIV status and how to enlist support from significant others during interactions with HIV positive pregnant women and mothers. Wherever possible, family-based and/or couple counselling should be provided to encourage close family members and partners to be involved in HEI testing. HCWs should also link HIV-positive women and mothers to peer support at health facilities and or in the community. These findings are consistent with the findings of many other studies that found that advice, experience sharing, and other forms of social support from peers increased the use of HEI testing services [28, 46–48].

### Individual level factors

#### Mother’s level of knowledge of HEI testing

The HIV-positive mothers in all the focus groups had a good understanding of PMTCT and HEI testing, though there were some gaps in knowledge about the types of tests and exact times for HEI testing. In all FGDs, many HIV-positive mothers expressed satisfaction with the PMTCT health education they received from HCWs, which motivated them to take their children to the health care facility for testing.

*"If you haven’t had your monthly periods in a while*, *you should see a doctor to check if it’s pregnancy or another illness*. *When you get there you do pregnancy tests and they will encourage you to check your HIV status*. *If you are already on ART*, *you will continue to take your treatment and they will teach you how to take your medication and when to attend your check-ups until you go for delivery*. *For me*, *I believe the education I received from nurses during my pregnancy*, *delivery*, *and afterward was sufficient and worked well for me*.*”*

*"When I was waiting to be discharged from the hospital after giving birth to my child*, *a certain nurse approached me and started assessing me*, *asking me about my welfare*, *where I live*, *who provides for us*, *and other household concerns*. *Some of the questions concerned my treatment*, *how I take it*, *and the challenges I face*. *She advised me on my treatment as well as the importance of testing my child*.*"*

On the other hand, some mothers were dissatisfied with the amount and quality of PMTCT education provided by HCWs and recommended that it should be improved. According to the mothers, the PMTCT education was there, but it was not comprehensive enough to enable them to make informed decisions about HEI testing. Some mothers reported having to rely on alternative sources of PMTCT information, such as the internet, family members, and peers. The mothers attributed the limited PMTCT education to staff shortages at health facilities and health education being provided by cadres of HCWs who they believed lacked the necessary expertise and training in PMTCT (lay counsellors, interns, and students).

*“For me it was difficult*. *They tested me and started me on treatment despite the fact that guidance from HCWs was insufficient*. *I ended up finding information from other sources and counselling myself*. *I was determined to do the right thing for my twins; I knew nothing about PMTCT and the HCWs only briefed me but I had to do my own research*.*”*

The mothers recommended that HCWs increase the frequency and depth of information that is provided to HIV-positive pregnant women and mothers to increase their understanding of HEI testing. The HCWs should not only explain what to do but also why it is necessary. Quality health education and counselling should be provided to HIV-positive pregnant women and mothers until all the recommended tests for HEI are completed. The mothers also advocated for increased community education about the importance of HEI testing through radio, home visits, Kgotla (community) meetings, churches, workplaces, and mobile phone technology.

These findings are consistent with previous research, which found that mothers who had a good understanding of PMTCT were more likely to access and use HEI testing services than mothers who did not [26–29, 48]. The findings show the need for the MOH to increase training for all HCWs who are involved in the implementation of the PMTCT program to strengthen their PMTCT health education skills. In accordance with the mothers’ recommendations, the MOH should consider using mobile phone technology to promote HEI testing. Community education campaigns using diverse approaches should reinforce the HEI testing education provided by HCWs in health care facilities.

#### Self-acceptance of HIV status by the mother

Self-acceptance by HIV-positive women was identified as a key motivator for HEI testing. Education and counselling from health care workers, as well as social support from a family member, child’s father, or trusted peer/friend, were identified as factors that aided the mothers’ acceptance of their HIV status. On the other hand, mothers’ lack of acceptance of HIV status was associated with inadequate HIV education and counselling, the mother being newly diagnosed with HIV, a lack of social support, and fear of stigma. The mothers advocated for increased counselling and education to support HIV-positive women to accept their status and to learn to live with it.

*"I went through it before I accepted myself*. *I was unable to test my child to the extent of losing him*. *After self–acceptance*, *I was able to test freely; I tested all my two children that were born after the death of the first one*. *I tested them so freely*.*”*

Other studies have found that poor self-acceptance of HIV status by the mother is a barrier to the utilization of HEI testing services [48]. Therefore, HIV-positive pregnant women and mothers should receive health education and counselling that emphasizes self-acceptance.

#### Child negligence or lack of responsibility for the child’s health

The majority of mothers in all the FGDs said that they took their children for testing at the recommended time because they wanted to know their children’s HIV status and ensure that if they were HIV positive, they would be started on ART at the appropriate time to avoid serious illness and mortality. However, the mothers said they knew other HIV-positive mothers in their communities who did not take their children for testing due to negligence or irresponsibility. Mothers in FGDs associated child neglect with risky lifestyles (alcohol abuse, excessive concern for fun and entertainment especially in young mothers, laziness, stubbornness), as well as a lack of knowledge of children’s rights by the mothers.

*"I managed by accepting responsibility as a mother and taking my child’s health seriously by following the doctor’s instructions and doing things on the stipulated dates*, *beginning with pregnancy up to the last test on the baby*. *I took care of the baby the way I was supposed to*. "

*“Some mothers spend more time drinking alcohol without having time to take care of their children or take them to the clinic for testing*.*”*

*“Some mothers are simply lazy to take their children for testing*, *others due to a love for entertainment*. *I have observed that*, *particularly among young mothers*, *they neglect their children’s health just because they are too preoccupied with entertainment*.*"*

To prevent individual circumstances or lifestyles from influencing HEI testing uptake, the mothers proposed that the government enact a policy requiring all HEI to be tested at the recommended time and those who are HIV positive to be initiated on ART immediately. If possible, all HEI should be tested at birth, while they are still in the ward, or when they come in for the six-week postpartum check-up. The mothers also proposed that in cases of child neglect, testing be performed, and legal action be taken against the negligent mother or caregiver. The mothers also advocated for community-wide initiatives to educate community members about children’s rights and the legal implications of child neglect.

*"The child has the right to life*. *There are indeed mothers who do not care at all about the welfare of their children*. *So the government of Botswana should do everything in its power to ensure that if a mother neglects their child*, *then something is done to that mother in order to save the child*. "

The mothers’ proposals are in line with the Botswana Children’s Act of 2009 which affirms the child’s right to life and good health [23]. The proposals are also in line with the United Nations Convention on the Rights of the Child which affirms the child’s right to “survival, life, and development” (Article 6), the right to health (Article 24) and the consideration of the best interests of the child in all the decisions made about the child [25]. These results call for raising awareness of children’s rights so that they are widely known by the HCWs, mothers/caregivers, and community members which may contribute to the understanding that HEI have a right to a timely diagnosis and thus promote HEI testing uptake.

#### Good adherence to ART during pregnancy and after delivery

Many mothers in the FGDs said they had confidence in the efficacy of PMTCT interventions, and as a result, they ensured good adherence to ART during pregnancy and after delivery. Those mothers said that they believed their children had a low risk of being HIV positive, which encouraged them to take the children for testing at the recommended time.

*“For me*, *it was easy*. *If you are told that you should take your treatment well so that you protect your child*, *it is a matter of taking that seriously*. *So for me*, *I took this information seriously*, *I made sure that I took the medicines on time*, *and things were easy for me when I tested my child; I did not have any fears*. *I think it would be difficult if I did not do the right thing*. *So as for me testing my child was never a problem because I knew I was taking my treatment properly*.*”*

*"As I was taking my child to the clinic for testing*, *I was telling myself that "my child is HIV negative*.*” I knew in my heart that I was taking my treatment correctly during pregnancy and there was nothing to fear*. *I went to the clinic*, *had him tested*, *and then waited for the results*. *Then I returned to the clinic and the results were negative*. *"*

*"It wasn’t easy*, *I tell you*. *They tested him when I was discharged from Princess Marina Hospital a few weeks after giving birth*, *but I never returned to get the results*. *I had the fear that my child might be HIV positive*. *This affected me*. *Every time I went for my child’s monthly weight check*, *I was reminded that the child needed to be tested*. *They even wrote on the card*, *and I would sometimes avoid taking him for a weight check*. *I wasn’t even ready when I finally took him for the test; I was just doing it for them*. *He was grown when I tested him*. *"*

This finding contradicts previous research from other African countries, which found that some mothers believed it was unnecessary to bring their children for testing because PMTCT interventions, such as strict adherence to ART, ensured their children were not at risk of HIV infection [39, 49, 50].The results emphasise the importance of providing intensive adherence support to all HIV-positive pregnant women and mothers to improve their understanding of the benefits of good ART adherence for both mother and child. Individualized, couple, and family-based support should be provided to identify and address the barriers to ART adherence.

#### A mother’s fear of an HIV-positive test result for the infant

The mothers’ fear of the possibility of their child testing HIV positive was found to be a barrier to HEI testing. Many mothers said that they were afraid to have their child tested because they were not prepared for the shock if the child tested HIV positive. Some mothers took their children for testing at the recommended time, but they feared to go back to the clinic to collect the results. The fact that PMTCT interventions are not completely protective (100%) and poor ART adherence during pregnancy were cited as the main sources of fear. There was also fear that if the child tested HIV positive, he or she would face stigma at home, school, and in the community. The mothers recommended that health care providers encourage HIV-positive mothers to seek peer support because advice from peers had helped some mothers gain self-acceptance, reduce fear, and encourage HEI testing.

*"It wasn’t easy*, *I tell you*. *They tested my child when I was discharged from Hospital a few weeks after giving birth*, *but I never returned to get the results*. *I had the fear that my child might test positive*. *This affected me*. *Every time I took my child to the clinic for his monthly weight check*, *I was reminded that the child needed to be tested*. *They even wrote on the card*, *and I would sometimes avoid taking him to the clinic for a weight check*. *I wasn’t even ready when I finally took him for the test; I was just doing it for them*. *He was grown when I tested him*. *"*

*"More sessions like this one*, *where HIV-positive mothers meet before 6 weeks*, *6 months*, *and 18 months*, *just like us*, *and are taught the importance of HEI testing*, *would be beneficial*. *Furthermore*, *invite those who have successfully tested their children and overcome all obstacles to share their experiences*.*"*

These findings are consistent with other studies which found that a mother’s fear of her child testing HIV positive is a barrier to HEI testing [19, 26, 27, 49]. Therefore, HIV-positive mothers must be adequately prepared for HEI testing through education and counselling, and their worries and fears about HEI testing must be identified and addressed during those sessions.

#### A mother or caregiver forgetting the child’s testing appointment

Some mothers reported forgetting to return the HEI for testing appointments due to hectic work schedules, being at school, being ill, preoccupation with stressful events, lack of reminders, and many other reasons. The mothers were often reminded by their partners, family members, or health care providers when they took their children to the health care facility for other CWC appointments. To address the problem of forgetting, the mothers proposed that health care providers help the mothers or caregivers of HEI in developing personal reminders. Health care providers should also use home visits or mobile phone technology to remind all mothers and caregivers of their children’s testing appointments.

*“For me*, *it was just forgetfulness; I did not keep the appointment because I had forgotten*. *I was reminded at the clinic when I had gone to check the weight of my child*. *I only tested at 2 months*. *If it was not for those nurses*, *I don’t know when I would have tested*. *At 6 months and 18 months*, *I also tested late because I had forgotten*. *I was still reminded by the nurses*. *Not that I had anything against testing*, *but it was a matter of forgetting*.*”*

*"The same way that you (Botswana-Baylor staff) tracked us who delivered in 2016 to come to this meeting*, *you should track us at 18 months to come and test our children*. *It appears that at 18months*, *we forget the testing*, *and I even forgot*, *so someone had to remind me*. *So if the health care providers could remind us*.*”*

These findings highlight the need for a multifaceted system to remind mothers of their children’s testing appointments and to fast-track lost-to-follow-up HIV-positive mothers and their infants.

### Strengths and limitations of the study

The study’s strength was that the sample was drawn from the three largest birthing sites in Botswana. The three study sites are referral facilities that serve women from a wide geographic area, including both urban and rural areas. To our knowledge, this was the first study in Botswana to use a qualitative approach (focus group discussions) to explore the perspectives of a large sample of HIV-positive mothers (142) on HEI testing. Only one qualitative study had used in-depth interviews to investigate the perspectives of HIV-positive mothers on the barriers to HEI testing in a sample of 21 mothers drawn solely from Gaborone [20]. As a result, far less was known about the scope and nature of the barriers and facilitators to HEI testing in Botswana. Therefore, the findings of this study provide a unique in-depth understanding of the perspectives of HEI testing service consumers, allowing for the development of appropriate interventions to promote HEI testing.

Despite its strengths, the study has limitations that should be addressed in future research. Using referral hospitals for sampling may have resulted in selection bias (that is, the participants included women who sought specialized services at referral facilities, and their experiences and perspectives may not be fully representative of other women who sought services at lower levels of the health care system). To minimize selection bias and maximize representativeness, the researchers used a maximum variation sampling strategy to ensure a sample of HIV-positive mothers with diverse social, economic, and clinical characteristics were included in the study. Furthermore, the study was conducted in 2019 to 2020 and explored HIV-positive women’s recall of HEI testing barriers and facilitators from 2016, so it is subject to recall bias. In addition, many mothers who participated in this study tested their children at the recommended time. As a result, the perspectives of mothers who did not take their children for testing at the recommended time are underrepresented. Future research should examine the perspectives of HIV-positive mothers who did not test their children at the right time.

Lastly, our study had the limitations inherent in qualitative research. For example, some mothers may perceive expectations to conform to social norms in the group setting. To mitigate this limitation, the facilitators created a safe environment in which all participants could express their views and share personal experiences without fear of blame or retribution. This included facilitators explaining the purpose of the study and laying ground rules for discussion, such as asking participants not to be judgmental and to treat one another’s views and perspectives with respect. However, the response effect may have persisted in some cases.

## Conclusion

The findings of this study provide a detailed description of HIV-positive mothers’ perspectives on the barriers and facilitators to HEI testing, as well as what needs to be done to eliminate the HEI testing deficit. The findings suggest that policy efforts and interventions to promote HEI testing should focus on health system strengthening to improve HEI testing service delivery. Increasing PMTCT program staffing, reducing stigma at health facilities, and strengthening PMTCT health education are key priorities for improving HEI testing. The findings also highlight the importance of prioritizing and addressing community barriers to HEI testing, such as bringing services closer to where people live, particularly in rural and hard-to-reach areas. A multidisciplinary and multi-level approach is needed to scale up the facilitators and address the barriers to HEI testing identified by this study.

### Recommendations for practice

Based on the perspectives of HIV-positive mothers in this study, the following actions are needed to optimise HEI testing in Botswana:

### 1. Intensify national-level strategies to promote HEI testing

Due to the multi-level barriers to HEI testing identified by the mothers in this study, the MOH should broaden mobilization, advocacy, and action to promote HEI testing in Botswana. Although the MTCT rate has decreased significantly, more efforts are needed to ensure that all HEI are identified and tested and that HIV-positive infants are started on ART as early as possible to avoid morbidity and mortality. All government and non-governmental organizations that provide institutional and community-based services to mothers and their children should be mobilized and encouraged to prioritize HEI testing. National-level surveillance data on HEI testing should be published on a regular basis to inform policy and programming in Botswana.

### 2. Strengthen the health care system to deliver more effective and efficient HEI testing services

Healthcare delivery issues were the most frequently mentioned barriers and facilitators to HEI testing. Therefore, the following actions should be prioritized by the health care system to improve HEI testing services:

#### i. Increase staffing levels in health facilities

According to the participants, the shortage of staff at health care facilities had a negative impact on the quality of PMTCT health education, limited the follow-up of mother-infant pairs, and resulted in multiple visits to the health care facilities for the mothers. Therefore, to improve service delivery, the MOH should increase the number of HCWs who are dedicated to providing PMTCT and HEI testing services. The PMTCT program (in collaboration with district health managers) should conduct a comprehensive workload analysis for all the cadres of HCWs providing PMTCT services. This will help in the development of realistic staffing standards for the PMTCT program.

#### ii. Expand the integration of HEI testing into other child health care services

The mothers in this study advocated for a consolidated one-stop-shop child health care service delivery model to reduce the number of visits to health care facilities, reduce the stigma associated with identifiable HIV testing services, as well as increase service delivery points for HEI testing. We recommend that HEI testing be fully integrated into child healthcare services at health facilities as well as community-based services for HIV-positive women and their infants. This will require providing standard guidelines to community-based workers in order for them to identify HEI that require testing.

#### iii. Increase PMTCT training for HCWs

The findings of this study emphasize the need to train HCWs in a range of areas to increase their PMTCT knowledge and skills and to enable them to provide effective services to clients. Therefore, we recommend that the MOH expand PMTCT training and retraining for all HCWs who provide PMTCT services, and the following areas should be prioritized:

- Training on DBS sample collection to enable more HCWs to provide HEI testing services.
- Instruction on how to use mobile phone technology to communicate PMTCT and HEI testing information to HIV positive mothers.
- Guidance on how to effectively and efficiently incorporate HEI testing into other child health care services, including guidelines on screening, identifying, and testing HEI.
- Educating HCWs on Botswana’s HIV testing policy for children and providing guidelines on how to handle various situations involving testing consent for children.

#### iv. Strengthen PMTCT health education

A mother having inadequate information on PMTCT was identified as a barrier to HEI testing. Therefore, the MOH (and partners) should implement the following measures to improve PMTCT health education:

- Recruit and increase the number of HCWs who are dedicated to providing PMTCT health education in health care settings.
- Train health care providers on effective methods for delivering PMTCT health education to HIV-positive women with varying educational levels.
- Consider using social media and information technology to educate HIV-positive women and other community members about the benefits of HEI testing.
- Ensure that PMTCT educational sessions include the following topics: importance of mothers’ self-acceptance of their HIV status; benefits of good ART adherence during pregnancy and after delivery; children’s rights; the benefits of HEI testing; where to take the HEI for testing; how to deal with the fear of testing the child; and involving significant others in HEI testing.

### 3. Enhance ART adherence support for all HIV-positive women during pregnancy and after delivery

Strict adherence to ART during pregnancy and after delivery gave HIV-positive mothers the confidence to test their children at the recommended time because they believed their children had a low risk of testing HIV positive. Therefore, all HIV-positive women should receive intensive adherence counselling sessions during ANC, PNC, and beyond. Peer support, as well as the use of mobile phone technology to promote ART adherence, should be considered.

### 4. The MOH should give high priority to rural and hard-to-reach settings by bringing the HEI testing services closer to where the people live

The most frequently reported barriers to HEI testing for women from rural and hard-to-reach communities were mother-infant pairs travelling long distances to health facilities with PMTCT services, challenges with transport money, and unreliable means of transport.

To overcome such barriers, there is a need to expand the availability of HEI testing services at local clinics, health posts, mobile stops, home-based testing, and other mobile outreach services. The MOH should engage community-based partners to support the delivery of HEI testing services in rural and hard-to-reach areas.

### 5. Intensify counselling and education to empower HIV-positive mothers to seek social support from family members, partners, and trusted peers or friends

According to the study findings, significant others have the potential to positively influence HEI uptake. Therefore, we recommend that the support needs of HIV-positive pregnant women and mothers be assessed on a regular basis through counselling and that potential sources of support be identified and utilized.

### 6. Improve HIV-positive women’s access to economic resources and financial independence

Lack of transportation money was a significant barrier to HEI testing, particularly for women living in rural and hard-to-reach areas. This could be improved if mothers were empowered to earn an income to cover transport costs and other financial obligations. Civil society organizations should play a role in supporting HIV-positive women access to employment, entrepreneurship, and other pathways out of financial constraints.

## Supporting information

S1 File Focus group discussion guide. (DOCX)
