# Supplementary material for: The barriers and facilitators of HIV-exposed infant testing as perceived by HIV-positive mothers in Botswana: A qualitative study
Source: PLoS One. 2022 Aug 31;17(8):e0273777. doi: 10.1371/journal.pone.0273777 (PMC9432723; doi:10.1371/journal.pone.0273777)
Supplement: S1 File — (DOCX) [file pone.0273777.s001.docx]

**TESTING OUTCOMES OF HIV EXPOSED INFANTS**

**HIV POSITIVE MOTHERS’PERSPECTIVES ON BARRIERS AND FACILITATORS TO HIV EXPOSED INFANT TESTING IN BOTSWANA**

**Focus Group Discussion Guide**

1. Introductions
2. Aims and Objectives of the study

**Discussions to assess the following:**

**Theme 1: Knowledge about Early Infant Diagnosis/ HIV Exposed Infant Testing**

1. Is it necessary for an HIV-positive mother to take her child for HIV testing?
2. When is the right time to take the child for HIV testing?
3. What are the benefits of testing the child early or at the recommended time?

**Theme 2: Experiences with Early Infant Diagnosis/ HIV Exposed Infant Testing**

1. Please tell us about your experiences with testing your child for HIV. Was it an easy or difficult task?
2. What makes other HIV-positive mothers or caregivers delay or fail to test their children at the recommended time?

**Theme 3: Concerns about Early Infant Diagnosis/HIV Exposed Infant Testing**

1. Please tell me about your disappointments with testing your child.

**Theme 4: Barriers and Obstacles to Early Infant Diagnosis/HIV Exposed Infant Testing**

1. Please tell me the challenges that you encountered that caused you to delay or not test your child.
2. What are the obstacles that make other people test their children late or when they are already sick?

**Theme 5: Enablers/ facilitators of HIV Exposed Infant Testing**

1. What do you think makes it easy for individuals, including yourself to successfully manage all the Early Infant Testing processes?
2. What would you advise or guide other HIV-positive mothers to do to ensure that their children are tested on time?

**Theme 6: Suggestions for improving Exposed Infant Testing**

1. What could be done to ensure that all infants born to HIV-positive mothers are taken to health care facilities for testing at the recommended time?
2. What can we all do to improve exposed infant testing?
3. What should healthcare workers do to improve exposed infant testing?
4. What should the government of Botswana do to improve exposed infant testing?

***Thank you so much for your time and for helping us find out what we can do to promote HIV Exposed Infant Testing in Botswana. Your experiences, perspectives and insights highlight the plight of thousands of other HIV positive mothers and their children in Botswana and the wider world. We are grateful for your participation.***
